# Prevalence and risk factors of dysphagia among nursing home residents in eastern China: a cross-sectional study

**DOI:** 10.1186/s12877-020-01752-z

**Published:** 2020-09-17

**Authors:** Shen Chen, Yan Cui, Yaping Ding, Changxian Sun, Ying Xing, Rong Zhou, Guohua Liu

**Affiliations:** 1 School of Nursing, Nanjing Medical University, 101 Longmian Avenue, Jiangning District, Nanjing, 211166 Jiangsu Province China; 2 Jiangsu Vocational Institute of Commerce, 180 Longmian Avenue, Nanjing, 211168 China; 3 JORU QingHe Senior Care Center, 70 Youyi Street, Nanjing, 210041 China

**Keywords:** Nursing homes, Dysphagia, Deglutition disorders, Prevalence, Risk factors, Aged, China

## Abstract

**Background:**

Dysphagia is a common health care problem and poses significant risks including mortality and hospitalization. China has many unsolved long-term care problems, as it is a developing country with the largest ageing population in the world. The present study aimed to identify the prevalence and risk factors of dysphagia among nursing home residents in China to direct caregivers towards preventative and corrective actions.

**Methods:**

Data were collected from 18 public or private nursing homes in 9 districts of Nanjing, China. A total of 775 older adults (aged 60 ~ 105 years old; 60.6% female) were recruited. Each participant underwent a standardized face-to-face interview by at least 2 investigators. The presence of risk of dysphagia was assessed using the Chinese version of the EAT-10 scale. The Barthel Index (BI) was used to evaluate functional status. Additionally, demographic and health-related characteristics were collected from the participants and their medical files. Univariate analyses were first used to find out candidate risk factors, followed by binary logistic regression analyses to determine reliable impact factors after adjusting for confounders.

**Results:**

Out of 775 older adults, the prevalence of dysphagia risk was calculated to be 31.1%. A total of 85.0% of the older adults reported at least one chronic disease, and diseases with the highest prevalence were hypertension (49.5%), stroke (40.4%), diabetes (25.5%) and dementia (18.2%). Approximately 11.9% of participants received tube feeding. The mean BI score was 56.2 (SD = 38.3). Risk factors for dysphagia were texture of diet (*OR* = 2.978, *p* ≤ 0.01), BI level (*OR* = 1.418, *p* ≤ 0.01), history of aspiration, pneumonia and heart attack (*OR* = 22.962, 4.909, 3.804, respectively, *p* ≤ 0.01), types of oral medication (*OR* = 1.723, *p* ≤ 0.05) and Parkinson disease (*OR* = 2.566, *p* ≤ 0.05).

**Conclusions:**

A serious risk of dysphagia was observed among Chinese nursing home residents. Overall, nursing home residents were moderately dependent, according to the BI level. The risk for dysphagia increased with thinner diet texture, worse functional status, history of aspiration, pneumonia and heart attack, more oral medications and Parkinson disease. The findings of our study may serve to urge nursing home staff to pay more attention to the swallowing function of all residents and to take more actions in advance to prevent or reduce dysphagia.

## Background

Dysphagia was recognized as a geriatric syndrome by the European Union Geriatric Medicine Society, who defined it as a condition involving perceived or real difficulty in forming or moving a bolus safely from the oral cavity to the oesophagus [1, 2]. In short, it is a difficulty in swallowing. Dysphagia has become one of the major health care problems in nursing homes, and it may lead to a decline in quality of life among many older residents [3]. The prevalence of dysphagia among nursing home residents in the literature varies as a result of screening or assessment tools used, participant selection, and different regions or countries. Dysphagia was reported in 13.4% of the residents from 19 counties in Europe and North America according to the polar question answered by nursing home staff [4]. In Italy and the Netherlands, studies have reported that 9.0–12.8% of nursing home residents suffered from dysphagia, as assessed by health care professionals, a standardized questionnaire or a resident’s response to a dichotomous question [5–7]. Using the Gugging Swallowing Screen test, a South Korean study described a prevalence of dysphagia of 52.7% in institutionalized older people [8]. Another study with older adults living in intermediate-care facilities in the Taiwan area found a prevalence of self-reported swallowing difficulty of 51.0% [9].

Dysphagia is a serious condition that may lead to many poor outcomes. Impaired swallowing efficacy or the inefficient ingestion of liquids and nutrients may cause dehydration and malnutrition [10, 11]. Impaired swallowing safety with airway invasion may lead to aspiration pneumonia, respiratory infections and sudden bolus death [10, 12, 13]. All these negative outcomes result in increased hospitalization, hospital readmission, psychological distress and mortality [1, 13, 14]. Although the complications of dysphagia can be severe, it is often undetected and untreated [15].

Dysphagia is more frequently a result of altered physiology of deglutition caused by ageing, frailty, cancer of the neck and oesophagus, and neurological diseases such as stroke, dementia and Parkinson disease [16–19]. A variety of methods are available to assess for dysphagia, with the gold standards being video fluoroscopic swallowing exam (VFSE) and fibreoptic endoscopic examination of swallowing (FEES) [20]. However, these methods are invasive techniques that may not be tolerated by every older resident and are generally not available in nursing homes. Hence, dysphagia is often assessed with non-invasive bedside swallowing tests, judgement by speech-language therapists and questionnaires or systematic interviews [1, 4, 21]. It was more convenient to use a questionnaire assessment in our study, as China’s nursing homes are usually daily life care institutions and rarely have medical services present.

China has the largest ageing population in the world, and the country is experiencing a rapidly increasing number of older adults who require long-term care. The National Bureau of Statistics of China reported that more than 249.5 million people were over 60 years old in 2018, approximately 17.9% of the total population of China [22]. Along with the accelerated prevalence of elderly people, the number of older adults with dysphagia is also increasing rapidly. Within the Confucian culture, most of older Chinese adults are cared for by their family members. Nevertheless, traditional caregivers have a much heavier burden than was previously the case, as there are increasingly more nuclear families caused by the “one child policy” and the rise of “female professionals” in China [23]. Consequently, there is an increase in older adults wanting to move into nursing homes. However, supply does not match demand as nursing homes in China are still at an early stage of development, with only approximately 7.5 million beds available in the whole country [24].

At present, prevalence data for dysphagia among nursing home residents in mainland China (main part of China other than special administrative regions) are not available. Most previous studies related to dysphagia in older adults were conducted in developed countries or regions [4–9]. Compared with other countries, China has a larger ageing population and a relatively less developed long-term care system, with different policies and lifestyles, so it is important to investigate the extent to which dysphagia is a problem in China. Therefore, the present study aimed to identify the prevalence and risk factors of dysphagia among older Chinese adults who live in nursing homes. The findings may be used to inform caregivers to establish an effective care programme for dysphagia.

## Methods

The protocol of this study was approved by Nanjing Medical University Ethics Committee (approval no.2019–917).

### Data source and analytic sample

A cross-sectional study design was used to collect data from a mix of 18 public and private nursing homes in 9 districts of Nanjing, Jiangsu Province. These nursing homes were located in rural, urban and suburban areas. The participants of this study were all residents living in these nursing homes who met the criteria for inclusion with a written agreement from themselves or their family members to participate in this study. The criteria for inclusion in the study were as follows: ≥60 years old; older adults had the ability to answer our questions or their caregivers who are familiar with their situation could provide answers; and did not have intellectual disabilities. The final sample yielded 775 older adults who met the inclusion criteria.

### Instruments

Three questionnaires were used to measure demographic and health-related information, functional status and risk of dysphagia:

1. Demographic and health-related questionnaire: This self-designed questionnaire was used to collect information on age, sex, education, marital status, chronic diseases that may cause dysphagia, body mass index (BMI), dental condition, muscle strength, aspiration history, pneumonia history, hospitalization history, texture of diet and types of oral medication. These variables were selected from previous studies [4–9] and suggestions from health care professionals. BMI and muscle strength were assessed by nurses, dental condition was assessed by dentists, and the other variables were collected from self-reports, main caregivers’ reports or medical files by nurses. We obtained agreement from the participants or their family members to use data within their medical files.

Dental condition included a check on the presence of normal teeth, artificial teeth and dental caries. Muscle strength was divided into 6 levels [25]: level 0 indicated that the limbs had no muscle contraction; level 1, limbs had muscle contraction but could not be intentionally moved; level 2, limbs could move in parallel in bed but could not move against gravity; level 3, limbs could lift away from the bed but cannot overcome resistance; level 4, limbs could not completely resist resistance; and level 5, muscle strength was normal. Aspiration history was defined as cough, dyspnoea or voice change while swallowing during the past month. Pneumonia history and hospitalization history were defined as diagnosed pneumonia or hospital transfer occurring during the past year. Texture of diet included general diet, soft diet, semi-liquid diet, liquid diet and tube feeding.

2. EAT-10 scale: This questionnaire was originally developed by Belafsky in 2008 [26], and translated into the Chinese version by an expert group in 2013; it is used for rapidly screening patients with dysphagia. It has 10 questions with the score of each ranging from 0 (no problem) to 4 (severe problem). The participants were defined as having dysphagia when the total score was ≥3. Although this method performs as a screening tool of dysphagia, it actually only determines the risk of dysphagia because it is not a gold standard.
3. Barthel Index (BI): This questionnaire was modified by Barthel and Mahoney in 1965 [27]; it is usually applied for measuring the dependency levels of basic activities of daily living (BADL), which include items of dressing, grooming, bathing, feeding, toileting, bladder control, bowel control, ambulating, chair/bed transfer and using stairs. The scores range from 0 to 100 as an indicator of the severity of the BADL disability. Scores are categorized into 5 levels: 100 (no dependency), 61–99 (mild dependency), 41–60 (moderate dependency), 20–40 (severe dependency), 0–19 (complete dependency) [27].

Chinese versions of the EAT-10 and BI showed good validity and reliability in previous studies [28, 29].

### Procedure

A 2-day training prior to commencement of the study was first provided to seventeen nursing professionals and post-graduate students to ensure competency in the data collection techniques. From May 2019 to July 2019, face-to-face interviews were carried out at 18 nursing homes with each participant, along with three separate questionnaires. Proxy versions of each questionnaire were available for caregivers of participants. All participants completed all interviews and questionnaires.

### Data analysis

Data analysis was conducted using the SPSS 24.0 software package. Frequencies were applied to describe the prevalence of risk of dysphagia and the disability status, as well as the characteristics of participants, along with means and standard deviations. Chi-square tests or t-tests were used, depending on normality of data to examine the association between dysphagia and particular characteristics. Eventually, binary logistic regression analyses were used to determine significant risk factors after adjusting for confounders.

## Results

### Characteristics

As Table 1 shows, of the 775 participants, 63.2% were aged 80 years or older. The mean age was 81.3 (SD = 9.3) years, and ages ranged from 60 to 105 years. More female (60.6%) than male residents (39.4%) were included, and unmarried residents (68.1%), which also included widowed or divorced residents, were more prevalent than married residents (31.9%). More than 50% had a minimum of a high school education. Approximately 67.6% could eat a general diet, but almost 12% needed to use feeding tubes; 64.6% had one or more dental caries. According to BMI measurements, 19.0% were underweight (BMI<18.5), and 30.9% were overweight (24 ≤ BMI<28) or obese (BMI ≥ 28). Approximately 11.4% of participants experienced aspiration during the past month. Pneumonia and hospitalization occurred in approximately 7.7 and 25.5% of participants, respectively, during the past year. The mean types of oral medication used per day by the participants was 2.5 (SD = 2.1), which ranged from 0 to 12 with a median of 2. The number of participants who had at least one chronic disease was 659 (85.0%), and diseases with the highest prevalence were hypertension (49.5%), stroke (40.4%), diabetes (25.5%) and diagnosed dementia (18.2%). The participants with mild cognitive impairment were not considered as dementia patients in this study.

**Table 1:** Demographic and health related characteristics of participants and results of the univariate analysis (*N* = 775)

Characteristics	Classification	Total (*n* = 775)	With dysphagia (*n* = 241)	Without dysphagia (*n* = 534)	*T/χ*^2^-Value	*p-*Value
Age (year)	(Mean ± SD)	81.3 ± 9.3	82.0 ± 9.5	81.0 ± 9.2	−1.410	0.159
	60 ~ 69 years	93 (12.0%)	30 (12.4%)	63 (11.8%)	1.920	0.383
	70 ~ 79 years	192 (24.8%)	52 (21.6%)	140 (26.2%)		
	≥80 years	490 (63.2%)	159 (66.0%)	331 (62.0%)		
Sex	Male	305 (39.4%)	101 (41.9%)	204 (38.2%)	0.956	0.328
	Female	470 (60.6%)	140 (58.1%)	330 (61.8%)		
Marital status	Unmarried	528 (68.1%)	148 (61.4%)	380 (71.2%)	7.270^**^	0.007
	Married	247 (31.9%)	93 (38.6%)	154 (28.8%)		
Education	Illiterate	206 (26.6%)	63 (36.1%)	143 (26.8%)	4.654	0.199
	Primary school	158 (20.4%)	42 (17.4%)	116 (21.7%)		
	High school	286 (36.9%)	88 (36.5%)	198 (37.1%)		
	College and above	125 (16.1%)	48 (19.9%)	77 (14.4%)		
Texture of diet	General diet	524 (67.6%)	74 (30.7%)	450 (84.3%)	332.421^**^	<0.001
	Soft diet	82 (10.6%)	19 (7.9%)	63 (11.8%)		
	Semi-liquid diet	28 (3.6%)	21 (8.7%)	7 (1.3%)		
	Liquid diet	49 (6.3%)	38 (15.8%)	11 (2.1%)		
	Tube feeding	92 (11.9%)	89 (36.9%)	3 (0.6%)		
BMI	(Mean ± SD)	22.0 ± 3.9	21.2 ± 4.0	22.4 ± 3.8	4.006^**^	<0.001
	Underweight (BMI<18.5)	147 (19.0%)	66 (27.4%)	81 (15.2%)	18.689^**^	<0.001
	Normal (18.5 ≤ BMI<24)	389 (50.2%)	115 (47.7%)	274 (51.3%)		
	Overweight(24 ≤ BMI<28)	188 (24.3%)	44 (18.3%)	144 (27.0%)		
	Obesity (BMI ≥ 28)	51 (6.6%)	16 (6.6%)	35 (6.6%)		
Dental condition	Normal teeth	136 (17.5%)	34 (14.1%)	102 (19.1%)	16.382^**^	<0.001
	Artificial teeth	138 (17.8%)	27 (11.2%)	111 (20.8%)		
	Dental caries	501 (64.6%)	180 (74.7%)	321 (60.1%)		
Aspiration history (Occurred during the past month)	No	687 (88.6%)	169 (70.1%)	518 (97.0%)	119.194^**^	<0.001
	Yes	88 (11.4%)	72 (29.9%)	16 (3.0%)		
Pneumonia history (Occurred during the past year)	No	715 (92.3%)	195 (80.9%)	520 (97.4%)	63.030^**^	<0.001
	Yes	60 (7.7%)	46 (19.1%)	14 (2.6%)		
Hospitalization history (Occurred during the past year)	No	577 (74.5%)	156 (64.7%)	421 (78.8%)	17.378^**^	<0.001
	Yes	198 (25.5%)	85 (35.3%)	113 (21.2%)		
Types of oral medication	(Mean ± SD)	2.5 ± 2.1	3.2 ± 2.3	2.2 ± 1.9	−5.756^**^	<0.001
	≤2	446 (57.5%)	107 (44.4%)	339 (63.5%)	24.757^**^	<0.001
	≥3	329 (42.5%)	134 (55.6%)	195 (36.5%)		
Dementia	No	634 (81.8%)	183 (75.9%)	451 (84.5%)	8.105^**^	0.004
	Yes	141 (18.2%)	58 (24.1%)	83 (15.5%)		
Parkinson disease	No	728 (93.9%)	215 (89.2%)	513 (96.1%)	13.701^**^	<0.001
	Yes	47 (6.1%)	26 (10.8%)	21 (3.9%)		
Stroke history	No	462 (59.6%)	99 (41.1%)	363 (68.0%)	49.904^**^	<0.001
	Yes	313 (40.4%)	142 (58.9%)	171 (32.0%)		
TBI history	No	763 (98.5%)	236 (97.9%)	527 (98.7%)	0.636	0.530^b^
	Yes	12 (1.5%)	5 (2.1%)	7 (1.3%)		
Cancer	No	752 (97.0%)	228 (94.6%)	527 (98.1%)	7.151^**^	0.007
	Yes	23 (3.0%)	13 (5.4%)	10 (1.9%)		
Diabetes	No	577 (74.5%)	177 (73.4%)	400 (74.9%)	0.187	0.666
	Yes	198 (25.5%)	64 (26.6%)	134 (25.1%)		
Hypertension	No	391 (50.5%)	118 (49.0%)	273 (51.1%)	0.310	0.578
	Yes	384 (49.5%)	123 (51.0%)	261 (48.9%)		
Hyperlipidemia	No	709 (91.5%)	228 (94.6%)	481 (90.1%)	4.376^*^	0.036
	Yes	66 (8.5%)	13 (5.4%)	53 (9.9%)		
Heart failure	No	731 (94.3%)	226 (93.8%)	505 (94.6%)	0.195	0.659
	Yes	44 (5.7%)	15 (6.2%)	29 (5.4%)		
Heart attack history	No	725 (93.5%)	218 (90.5%)	507 (94.9%)	5.540^*^	0.019
	Yes	50 (6.5%)	23 (9.5%)	27 (5.1%)		
COPD	No	760 (98.1%)	236 (97.9%)	524 (98.1%)	0.036	1.000^b^
	Yes	15 (1.9%)	5 (2.1%)	10 (1.9%)		
Digestive diseases	No	734 (94.7%)	224 (92.9%)	510 (95.5%)	2.171	0.141
	Yes	41 (5.3%)	17 (7.1%)	24 (4.5%)		

*BMI* Body Mass Index, *TBI* Traumatic Brain Injury, *COPD* Chronic Obstructive Pulmonary Diseases

^a^ Some residents have no drugs, so the total number is less than 775

^b^ Use adjusted Chi-square analysis

^*^ *p* ≤ 0.05; ^**^p ≤ 0.01

### Disability status

As Table 2 shows, the mean score of BI was 56.2 (SD = 38.3); therefore, overall, the participants were moderately dependent on BI level. The participants with mild dependency held the highest percentage (29.3%), followed by complete dependency (24.6%) and no dependency (21.8%). There were 51.1% of participants classified with varying degrees of decline in muscle strength, and of these, 10.3% were classified as level 0, which indicated that their limbs had no muscle contraction.

**Table 2:** Disability status of participants and results of the univariate analysis (*N* = 775)

Variables	Classification	Total (*n* = 775)	With dysphagia (*n* = 241)	Without dysphagia (*n* = 534)	*T/χ*^*2*^	*p-*Value
BI	(Mean ± SD)	56.2 ± 38.3	24.6 ± 30.8	70.4 ± 32.4	18.506^**^	<0.001
	No dependency (100 points)	169 (21.8%)	9 (3.7%)	160 (30.0%)	233.516^**^	<0.001
	Mild dependency (61 ~ 99 points)	227 (29.3%)	28 (11.6%)	199 (37.3%)		
	Moderate dependency (41 ~ 60 points)	81 (10.5%)	26 (10.8%)	55 (10.3%)		
	Severe dependency (20 ~ 40 points)	107 (13.8%)	43 (17.8%)	64 (12.0%)		
	Complete dependency (0 ~ 19 points)	191 (24.6%)	135 (56.0%)	56 (10.5%)		
Muscle strength	Level 0 (Zero)	80 (10.3%)	52 (21.6%)	28 (5.2%)	134.669^**^	<0.001
	Level 1 (Trace)	38 (4.9%)	23 (9.5%)	15 (2.8%)		
	Level 2 (Poor)	78 (10.1%)	44 (18.3%)	34 (6.4%)		
	Level 3 (Fair)	60 (7.7%)	22 (9.1%)	38 (7.1%)		
	Level 4 (Good)	140 (18.1%)	47 (19.5%)	93 (17.4%)		
	Level 5 (Normal)	379 (48.9%)	53 (22.0%)	326 (61.0%)		

*BI* Barthel Index

^**^p ≤ 0.01

### Prevalence of dysphagia risk

As shown in Table 3, the mean EAT-10 score was 5.5 (SD = 10.7), and the prevalence rate of risk of dysphagia was 31.1%. Some symptoms and signs of dysphagia were reported by the participants. Difficulty in swallowing solids occupied the highest percentage (29.5%), followed by coughing while/after swallowing (28.6%) and difficulties in swallowing pills (27.2%). Other symptoms and signs were observed in a lower percentage of participants.

**Table 3:** Symptoms and signs of dysphagia among nursing home residents (*N* = 775)

Symptoms and signs	*n*	%
Scores of EAT-10 (Mean ± SD)	5.5 ± 10.7	
Dysphagia (EAT-10 ≥ 3)	241	31.1
Weight loss	168	21.7
No ability to go out for meals	163	21.0
Difficulty in swallowing liquids	184	23.7
Difficulty in swallowing solids	229	29.5
Difficulty in swallowing pills	211	27.2
Pain from swallowing	129	16.6
Less pleasure when eating	173	22.3
Food sticking in the throat	170	21.9
Coughing	222	28.6
Stress from swallowing	137	17.7

### Risk factors associated with dysphagia

Univariate comparisons that used with/without dysphagia as the dependent variable and dependency levels, demographic characteristics and health-related characteristics as independent variables were undertaken. As shown in Tables 1 and 2, the results indicated that marital status (*χ*^*2*^ = 7.270), texture of diet (*χ*^*2*^ = 332.421), BI level (*χ*^*2*^ = 233.516), muscle strength level (*χ*^*2*^ = 134.669), BMI level (*χ*^*2*^ = 18.689), dental condition (*χ*^*2*^ = 16.382), aspiration history (*χ*^*2*^ = 119.194), pneumonia history (*χ*^*2*^ = 63.030), hospitalization history (*χ*^*2*^ = 17.378), types of oral medication (*χ*^*2*^ = 24.757), dementia (*χ*^*2*^ = 8.105), Parkinson disease (*χ*^*2*^ = 13.701), stroke history (*χ*^*2*^ = 49.904), cancer (*χ*^*2*^ = 7.151), hyperlipidaemia (*χ*^*2*^ = 4.376) and heart attack history (*χ*^*2*^ = 5.540) were significantly associated with dysphagia. T-test results revealed a significant association between dysphagia and BI scores, BMI values, and types of oral medication (*T* = 18.506, 4.006, − 5.756, respectively, *p* ≤ 0.01).

Binary logistic regression analysis, which used with/without dysphagia as the dependent variable and significant impact factors from the univariate comparisons as independent variables, was undertaken to identify the final risk factors associated with dysphagia. All listed variables were adjusted by the regression model. Table 4 shows that the likelihood of dysphagia increased with thinner diet texture (*OR* = 2.978, *p* ≤ 0.01), worse disability BADL status (*OR* = 1.418, *p* ≤ 0.01), history of aspiration, pneumonia and heart attack (*OR* = 22.962, 4.909, 3.804, respectively, *p* ≤ 0.01), more oral medications (*OR* = 1.723, *p* ≤ 0.05) and Parkinson disease (*OR* = 2.566, *p* ≤ 0.05).

**Table 4:** Risk factors of dysphagia among nursing home residents-results of binary logistic regression (*N* = 775)

Variables	*B*	*p-*Value	Adjusted *OR*	95%C.I. for *OR*	
				Lower	Upper
Marital status	0.141	0.607	1.152	0.672	1.973
Texture of diet	1.094	<0.001	2.978^**^	2.378	3.751
BI	0.349	0.002	1.418^**^	1.139	1.766
Muscle strength	<0.001	0.998	1.000	0.841	1.189
BMI	−0.088	0.560	0.916	0.680	1.232
Dental condition	−0.027	0.874	0.974	0.701	1.353
Aspiration history	3.134	<0.001	22.962^**^	11.142	47.321
Pneumonia history	1.591	0.001	4.909^**^	1.855	12.991
Hospitalization history	0.284	0.312	1.328	0.766	2.304
Types of oral medication	0.544	0.038	1.723^*^	1.031	2.880
Dementia	0.175	0.572	1.192	0.649	2.187
Parkinson disease	0.942	0.047	2.566^*^	1.012	6.511
Stroke history	0.232	0.383	1.261	0.749	2.122
Cancer	1.359	0.052	3.893	0.988	15.342
Hyperlipidemia	−0.765	0.129	0.465	0.173	1.251
Heart attack history	1.336	0.002	3.804^**^	1.636	8.845

*BI* Barthel Index, *BMI* Body Mass Index

^*^ *p* ≤ 0.05; ^**^p ≤ 0.01

## Discussion

This cross-sectional study aimed to identify the prevalence and risk factors of dysphagia among nursing home residents in China to direct caregivers towards preventative and corrective actions. There was a high participation rate in this study, although some participants did have difficulties answering some questions; in such cases, their main caregivers or medical files could provide the needed information. Therefore, the results of this study could reveal the general characteristics of nursing home residents in a certain area of China. The data revealed a much lower prevalence of dementia in China’s nursing homes than that in western countries’ nursing homes [5]. The reason may come from that many older Chinese people with dementia prefer to live in their own homes rather than live in nursing homes. We also found that the proportion of participants who had at least a high school education, was higher than the percentage of 39.2% that had been found in previous studies carried out in community-based samples [30]. Although an increasing number of nursing homes are being established in China, many Chinese people who are influenced by the traditional confusion lifestyles are reluctant to accept them [31]. For example, perceptions of abandonment may prevail among children whilst older adults may not want to be transferred to an unfamiliar environment [31, 32]. Furthermore, older adults may incur much higher out-of-pocket costs by using a nursing home [33], as most of them do not have long-term care insurance; conversely those with higher education are more likely to be able to afford the costs. The data revealed that only 67.6% of residents could eat a general diet, and of the remainder, almost 12.0% needed to use feeding tubes. The rate of feeding tube use was slightly lower that the 16.4% reported in a 19-country study [4]. Previous studies have shown that patients with acute dysphagia who have a favourable prognosis are encouraged to use feeding tubes because they are useful in reducing nutritional complications [34]. However, no evidence supports the notion that tube feeding prevents aspiration [12, 34]. Moreover, there are several studies that recommended avoiding tube feeding in patients with advanced dementia as this intervention was associated with increased morbidity and mortality in this population [12, 34–36]. In China’s nursing homes, most of the staff are care workers with relatively low levels of medical knowledge, and so the few available nurses become the primary and often sole health care providers [37]. Registered health professionals are so limited in number that they may use them simply for their convenience in feeding older adults with dysphagia and avoiding aspiration. Hence, it is important that nursing home managers review the skills mix of their health care staff and provide more training for care workers on methods to deal with and recognize dysphagia, such as bolus modifications and appropriate swallowing posture and manoeuvres [1, 16, 38].

In this study, a prevalence rate of 31.1% for the risk of dysphagia among nursing home residents in mainland China was identified. This prevalence rate was higher than that found in nursing home studies in Europe and North America, which ranged from 9.0 to 13.4% [4–7]. However, the rate is lower than the prevalence rate of 51.0% ~ 52.7% found in nursing home studies in South Korea and Taiwan area [8, 9]. Different lifestyles and attitudes may explain the large gap between the rates in the West and the East. As previously mentioned, the lifestyles of East Asian countries usually urge older adults to live their later years of life around their children, especially sons [30]. It is usually unacceptable for older adults to live far away from their relatives [31]. Consequently, it is only if older people suffer from severe problems that are difficult to manage by their family members, for example, dysphagia, that help may be sought from nursing homes. This may account for the higher prevalence of dysphagia found in nursing home residents from Eastern countries or regions.

However, as a developing country, China is also different from other developed areas of Eastern Asia. The cost of care in nursing homes is high in China, according to the data published by the National Bureau of Statistics of China, the average income per month was 2352 yuan (approximately 334 US dollars) among the Chinese population in 2018 [22]. However, nursing homes usually charge more than 5000 yuan per month, which is a large burden for most older adults. Some elderly individuals with dysphagia are willing to live in a nursing home, but instead they remain at home as they cannot afford the nursing home costs [31]. Therefore, this might be one reason why, compared with other studies conducted in Eastern Asia, China had a lower prevalence of dysphagia.

Another reason for the difference in prevalence rates for dysphagia in the literature is the variety of screening or assessment tools. The studies that used subjective screening methods usually reported a lower prevalence of dysphagia than those that used objective assessment tools [5–9, 39]. Objective tools are more precise in identifying unnoticed or hidden dysphagia [8]. However, the resources and abilities of nursing home staff should also be taken into consideration when selecting tools. Sometimes, questionnaires are a quick and convenient tool in screening elders with a risk of dysphagia, especially in nursing homes with little medical equipment, so that timely interventions can be applied to older adults. In fact, this was the reason why, in this study, questionnaires were used.

To identify the risk of dysphagia early and properly prevent it, these findings suggest that nursing home staff should consider the risk factors of dysphagia in those residents who have difficult performing activities of daily living. Binary logistic regression analyses revealed that the BADL disability status was significantly associated with a higher risk of dysphagia, which was consistent with several previous studies. Sarabia-Cobo et al. performed a follow-up study in 2384 elderly Spanish patients, and they found that participants with dysphagia showed lower functional status [40]. Park et al. found that severely dependent functional status was one of the risk factors associated with dysphagia in Korean nursing home residents [8]. In a Dutch study, the care dependency scale score was associated with the presence of subjective dysphagia [6]. Hence, nursing home staff should assess, more frequently, swallowing function in older adults with severe levels of ADL dependency. The available evidence indicates that stroke and dementia are common medical conditions that can result in dysphagia [41–44]. However, in contrast to previous studies, we found that the association between dysphagia and dementia or stroke disappeared after adjusting the binary logistic regression. One reason for this unexpected finding may be the dependency level of participants. In most cases, stroke or dementia can increase dependency levels [45, 46], but elderly individuals who are well rehabilitated after stroke or with mild dementia may have little or no dependency on others, so they may not have the problem of dysphagia. Thus, swallowing disorders caused by dementia and stroke result from a decline in functional status. With regard to those conditions that increase the risk of dysphagia found in this study, several other studies have shared the same conclusion that Parkinson disease and heart attack history could increase the risk of dysphagia. Miller et al. reported that 80% of patients with Parkinson disease showed a slower swallowing rate than healthy controls [47]. Van der Maarel-Wierink et al. indicated that the disease cluster ‘cardiovascular disease’ was a significant variable in the multivariate backward stepwise regression analysis [6].

Moreover, frequent aspiration and pneumonia have been proven to be complications of dysphagia [10, 12], but they can also be used as predictors of swallowing disorders. Their presence may indicate that swallowing disorders have progressed to a more severe level. Findings from the current study also revealed that particular texture of diet and types of oral medication were associated with a higher risk of dysphagia. Daily meals for older adults are usually prepared by professional chefs, and some nursing homes provided a wide variety of food options. If older adults showed a preference for soft diet, semi-liquid diet or liquid diet, nursing home staff should more closely observe them to determine whether swallowing difficulties are present. Furthermore, older adults taking more types of oral medication were more likely to suffer from dysphagia, however this finding may have resulted from a decline in overall health [48]. Among the total population, 27.2% reported having difficulty swallowing pills. Thus, this in addition to a preference for a soft diet could have caused a negative cycle to develop in these older adults. This suggests that nursing home staff should assess various symptoms and signs that increase the risk of dysphagia, especially during meal and medication times.

It must be noted that this study has some limitations. First, this is a cross-sectional study, which restricts the ability to catch the causal relationship among the variables. Second, some of our data were collected by participants’ self-reporting, which may have led to information bias, as some participants may have concealed their real behaviour. Third, our samples of older adults were recruited from a relatively wealthy region of China, which may have resulted in selection bias. The fourth was the fact that this study only looked at the risk of dysphagia, rather than conducting a comprehensive assessment of swallowing. Future studies are needed to recruit older people from different regions of China and apply a more objective and comprehensive assessment of the swallowing method rather than only a questionnaire investigation.

## Conclusions

In this study, a serious risk of dysphagia has been observed among Chinese nursing home residents, with a prevalence rate of more than 30%. Nursing home residents were moderately dependent on basic activities of daily living overall. Dysphagia increased with thinner diet texture, worse functional status, history of aspiration, pneumonia and heart attack, more oral medications and Parkinson disease. The key recommendation from the findings of this study is to urge nursing home staff to pay more attention to the swallowing function of all residents and to take more actions in advance to prevent or reduce dysphagia.

## Data Availability

Our data may not be shared directly, because it is our team work, informed consent should be attained from all the team members. Our data or material may be available after contacting corresponding author or first author.
